# Tailoring the Dielectric Layer Structure for Enhanced Performance of Organic Field-Effect Transistors: The Use of a Sandwiched Polar Dielectric Layer

**DOI:** 10.3390/ma9070545

**Published:** 2016-07-07

**Authors:** Shijiao Han, Xin Yang, Xinming Zhuang, Junsheng Yu, Lu Li

**Affiliations:** 1 State Key Laboratory of Electronic Thin Films and Integrated Devices, School of Optoelectronic Information, University of Electronic Science and Technology of China (UESTC), Chengdu 610054, China; hanshijiao@outlook.com (S.H.); xmzhuang@std.uestc.edu.cn (X.Z.); 2 Co-Innovation Center for Micro/Nano Optoelectronic Materials and Devices, Research Institute for New Materials and Technology, Chongqing University of Arts and Sciences, Chongqing 402160, China; yangx@cqwu.edu.cn

**Keywords:** organic field-effect transistors (OFETs), dielectric/semiconductor interface, polar dielectric material, hydroxyl group

## Abstract

To investigate the origins of hydroxyl groups in a polymeric dielectric and its applications in organic field-effect transistors (OFETs), a polar polymer layer was inserted between two polymethyl methacrylate (PMMA) dielectric layers, and its effect on the performance as an organic field-effect transistor (OFET) was studied. The OFETs with a sandwiched dielectric layer of poly(vinyl alcohol) (PVA) or poly(4-vinylphenol) (PVP) containing hydroxyl groups had shown enhanced characteristics compared to those with only PMMA layers. The field-effect mobility had been raised more than 10 times in *n*-type devices (three times in the *p*-type one), and the threshold voltage had been lowered almost eight times in *p*-type devices (two times in the *n*-type). The on-off ratio of two kinds of devices had been enhanced by almost two orders of magnitude. This was attributed to the orientation of hydroxyl groups from disordered to perpendicular to the substrate under gate-applied voltage bias, and additional charges would be induced by this polarization at the interface between the semiconductor and dielectrics, contributing to the accumulation of charge transfer.

## 1. Introduction

Organic field-effect transistors (OFETs) have become of great interest for several electronic applications, such as active-matrix flat panel displays [1,2], electronic paper [3,4,5], and chemical sensors [6,7,8,9], due to their potential advantage in transparent and flexible devices. Among many possible performance impact factors, the overall performance of an OFET critically depends on the choice of the gate dielectrics [10]. Polymeric insulators have been considered as a gate dielectric material due to having advantages over inorganic ones [11,12]: (i) solution processibility, which can lower the fabrication cost and enable large-scale OFETs, and (ii) excellent flexibility on the plastic substrates, for example. The reason for the experimentally-obtained lower field-effect mobility compared to the ideal maximum value is believed to be due to the poor crystallinity of the semiconducting layer [13,14], the weak intermolecular bonding nature [15,16], and interface characteristics [17,18]. These indicate that there is still room for further improvement by optimizing the surrounding materials and processes used in the fabrication of OFETs [19].

When used as the gate dielectric, polymers with hydroxyl groups (such as the poly(vinyl alcohol) (PVA) and poly(4-vinyl phenol) (PVP)) present pronounced hysteresis in transfer characteristics [20]. Lee et al. reported that the increase of hydroxyl groups in polymer dielectric resulted in an equal increase in the number of trapping sites at the semiconductor/dielectric interface, which caused a large hysteresis [21]. Im et al. and Orgiu et al. demonstrated hysteresis-free OFETs by applying PVP and PVA dielectrics with cross-linkers and photosensitizers, respectively [22,23]. Additionally, as reported by Egginger et al., the thermally-activated motion of ionic impurities in PVA dielectric under the influence of applied electrical fields could serve as another parameter to modulate the transfer characteristics of OFETs [24]. More interesting, Ukah et al. reported that a high dipole moment solvent could help organize the polymer dielectric chains (PVP and PMMA) improving the semiconductor-insulator interface, which leaded to low hysteresis, low operating voltages, and reduced threshold voltages [25,26]. The above results indicate that manipulating the hydroxyl groups of PVA or PVP dielectrics to improve the performance of OFETs is a rather complicated process.

It is well known that PVA and PVP are polar polymers, and the polarity of PVA and PVP results from the electrostatic dipoles of hydroxyl groups, due to the different levels of electronegativity associated with oxygen and hydrogen atoms. The polarization of hydroxyl groups, such as the orientation or alignment of the dipoles, could be changed by variation of the electrical field (bias). Previous study showed that the applied gate bias partially aligns the orientations of hydroxyl groups perpendicular to the substrate (or parallel to the electrical field) [20]. Hence, the orientation modulation of hydroxyl groups in the PVA dielectric has been widely used in OFETs memory elements. However, the study to improve the performance of OFETs by utilizing the groups was rarely reported.

Nimmakayala et al. reported a concept fabrication method using a trilayer dielectric system that contained a PVA or PVP layer. The performance and stability of OFETs were improved [27], but the third layer Al_2_O_3_ was grown by converting a portion of Al film by an anodization process, even the annealing temperature of the solution process Al_2_O_3_ was at least 150 °C [28], which was not compatible with plastic substrates [29]. In this work, we demonstrate a trilayer dielectric system consisting of a polar dielectric layer inserted between hydrophobic polymer layers, which is fabricated with a solution process. The hydrophobic layer on the polar dielectric will separate hydroxyl groups from the conducting channel. Thus, the hydroxyl groups will not trap charge carriers directly but form an induced field to enhance the density of carriers at the semiconductor/dielectric interface. In order to demonstrate this effect, we have fabricated the OFETs with pentacene, α-sexithiophene (α-6T), and poly(3-hexylthiophene-2,5-diyl) (P3HT), as organic *p*-type semiconductor materials, and *N*,*N*′-Dioctyl-3,4,9,10-perylenedicarboximide (PTCDI-C8) as organic *n*-type semiconductor materials. PMMA, PVA, and PVP were used as the dielectric materials, where PVA and PVP contain polar hydroxyl groups.

## 2. Results and Discussion

**Figure 1:**
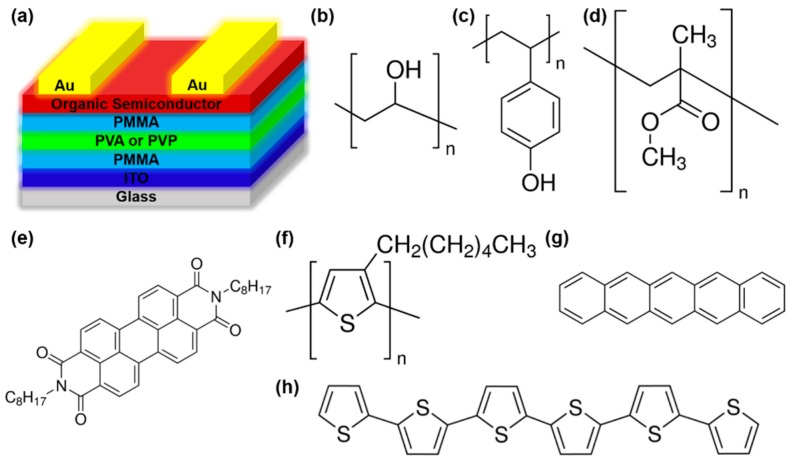
(**a**) Schematic structure of OFET with polymer trilayer dielectric, along with molecular structures of (**b**) PVA; (**c**) PVP; (**d**) PMMA; (**e**) PTCDI-C8; (**f**) P3HT; (**g**) pentacene; and (**h**) α-6T.

The architecture of the OFETs with trilayer dielectrics is shown in Figure 1, along with the chemical structures of the polymer dielectrics and organic semiconductor used in this work.

**Figure 2:**
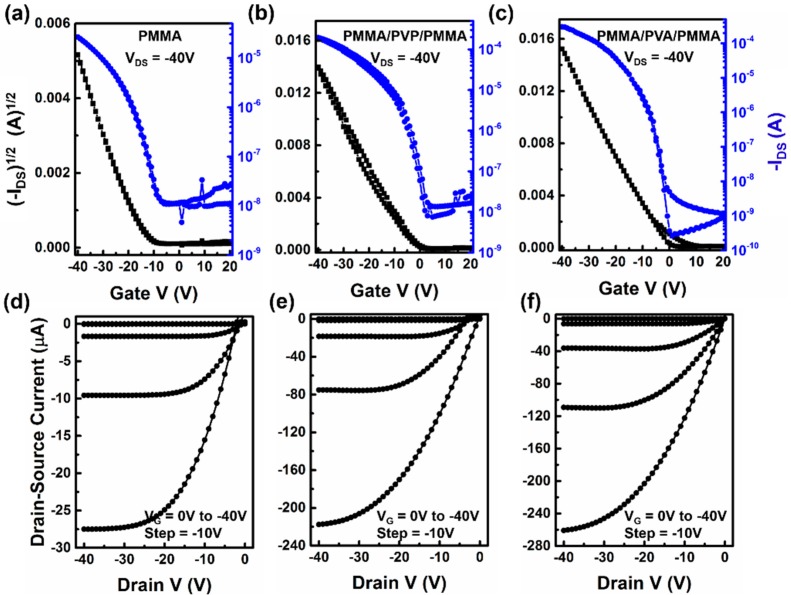
Typical transfer (*V_DS_* = −40 V) and output (*V_G_* = 0 to −40 V, with a −10 V step) characteristics of pentacene-based OFETs with different dielectric structure. (**a**,**d**) PMMA; (**b**,**e**) PMMA/PVP/PMMA; (**c**,**f**) PMMA/PVA/PMMA.

The transfer characteristics of OFETs were operated in forward (20 to −40 V) and backward (−40 V to 20 V) scans of *V_G_* in successive steps of 1 V, with a fixed *V_DS_* of −40 V. Figure 2a–c depict the representative transfer plots of the OFETs with different dielectric structures prepared from pentacene. The conference device and devices with the trilayer dielectric system containing PVP and PVA were named A, B, and C. Not unexpectedly, all devices demonstrated typical *p*-type transistor characteristics, and almost no hysteresis was found in the forward and backward scans of transfer characteristics in Figure 2b,c. Figure 2d–f show the output characteristics curve (*I_DS_* versus *V_DS_*) of devices A, B, and C, respectively. The devices showed clear linear and saturation behaviors below *V_DS_* = −20 V, and device A can be operated within −30 V. However, devices B and C could not be operated to saturation until −35 V. The field-effect mobility (*µ*), current on/off ratio (*I_on_*/*I_off_*), threshold-voltage (*V_T_*), and sub-threshold slope (*SS*) of different devices are shown in Table 1.

It can be clearly found that the devices with the PVA dielectric layer exhibited the best performance. The saturation current of device C was almost ten times higher than that of device A. The observed increase of current during bias stress in OFETs can be ascribed either to dielectric polarization or to charge injection from the gate [30]. Since the maximum gate current had not significantly changed in our devices (<10^−9^ A, see in Figure S1), charge injection from the gate could be ruled out, so polarization effects must be the main reason for the observed drain-current increase. Such polarization may be enhanced by the presence of highly-ordered dipolar groups in the polar dielectric under gate bias stress. After applying bias stress, the orientation of the dipolar groups, with respect to the applied gate field, may change, thus maximizing the polarization, which would explain why the output currents of devices B and C needed higher drain-source voltage to reach saturation than that of device A (shown in Figure 2d–f). Thus, the saturation currents of the devices are highly dependent on the polar dielectric layers.

After introducing a PVA dielectric layer, the *V_T_* had shifted from −16 to −2 V. Since V_T_ is usually referred to charge trapping at the semiconductor/dielectric interface, a more positive position means more charge carriers accumulated. Thus, there must be more hole-charges accumulated in device C than device A. As a result, the *μ* of device C was about 0.51 cm^2^V^−1^s^−1^, almost three times higher than that of device A (0.17 cm^2^V^−1^s^−1^). Furthermore, *SS* is proportional to the trap density at the semiconductor/dielectric interface, and the trap density (N) can be obtained by Equation (1) [31]:

(1) $$SS=\frac{kT}{q}\log_{10}(1+\frac{qN}{c})$$

where *q* is the electronic charge, *k* is Boltzmann’s constant, *T* is absolute temperature, and *c* is the areal capacitance of the dielectric structure. Thus, *N* changes in direct proportion to *SS*. As shown in Table 1, *SS* decreased to 1.5 V/dec in device B, and 3.8 V/dec in device C, respectively. It meant that trap density in devices B and C were lower than that in device A, so the hydroxyl groups did not contact the accumulation layers directly [32].

**Figure 3:**
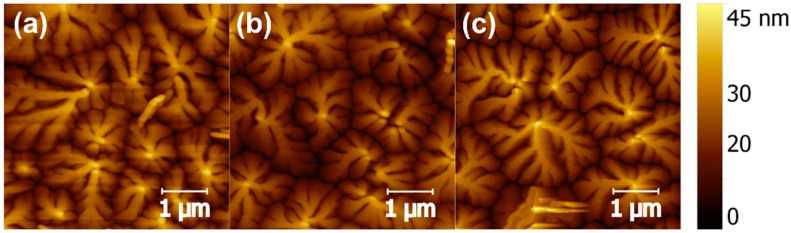
AFM height images of pentacene films deposited on three kinds of dielectics, (**a**) device A; (**b**) device B; and (**c**) device C.

Figure 3 presents AFM height images of pentacene films deposited on different dielectric structures. The surface morphologies, grain sizes and boundaries, and the roughness of pentacene films for these three samples were quite similar. Therefore, the differences in the intrinsic and electrical properties of the dielectric layers were responsible for variation in devices A, B, and C. The accumulation of charges on the gate dielectric layer or at the semiconductor/dielectric interface was critical to modulate the performance of the OFETs with polar dielectric layers.

Since PVA and PVP are polar polymers with abundant hydroxyl groups attached to polymer chains, the polarizations of hydroxyl dipoles would be sensitive to the magnitude and direction of the electrical field (bias) [27]. The influence of applied gate bias on the orientation of hydroxyl groups in the PVA layer was studied by Tsai et al. [20]. They found that the electrical field (pre-bias) had no influence on modulating the average orientations of the C–H bonds in PVA, but the average orientations of hydroxyl groups in the PVA film were subject to change with the applied electrical field. Presumably, the hydroxyl groups will be aligned perpendicular to the substrate under applied gate voltage (−40 V). Insulated by PMMA, the hydroxyl groups of PVA and PVP do not contact the transport channel directly, so they do not trap charge carriers, but can induce plenty of hole-carriers at the pentacene/dielectric interface. As a result, *μ* increases significantly, and *V_T_* shifts to the positive direction. For increasing charges in the accumulation layer, the saturation current increases, and additional voltage should be applied on the drain and source to drive the extra carriers. That is the reason that devices B and C should have higher *V_DS_* applied in order to reach the saturation region than that of device A.

**Figure 4:**
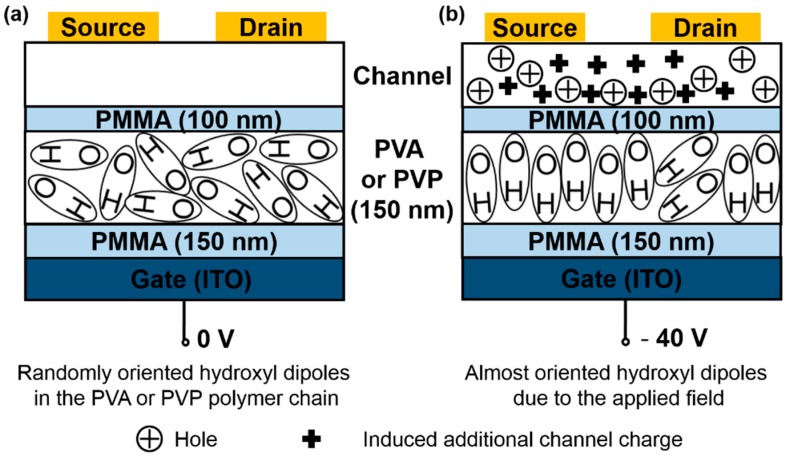
(**a**) Schematic illustration of disorder hydroxyl groups in polar dielectric layer; and (**b**) oriented hydroxyl groups due to applied gate voltage.

To illuminate the behavior of hydroxyl group orientation and bias-stress clearly, hypothesis analysis is performed, and the schematic illustration is presented in Figure 4. It can be seen that, as shown in Figure 4a, under 0 V, the hydroxyl groups attached to polymer chains are randomly oriented within the PVA or PVP layer. When the gate bias is switched on, as shown in Figure 4b, the hydroxyl groups begin to orient along the gate-field direction and the maximum polarization occurs when the *V_G_* reaches its maximum of −40 V. If there is no PMMA between the polar dielectric layer and semiconductor/dielectric interface, the oriented hydroxyl groups would promote charge trapping or electrostatically stabilize the accumulated charges at the interface. As a result, the threshold voltage shifts toward the negative position, and the hysteresis in transfer characteristics at the backward scan appears. However, in the present case, insulated by PMMA, the oriented hydroxyl groups produce an external field which will induce more charges at the dielectric/semiconductor interface. During the process of hydroxyl groups getting aligned along the gate field, additional charges keep inducing and contributing to charge transport. Therefore, the drain current increases, as shown in Figure 2.

**Figure 5:**
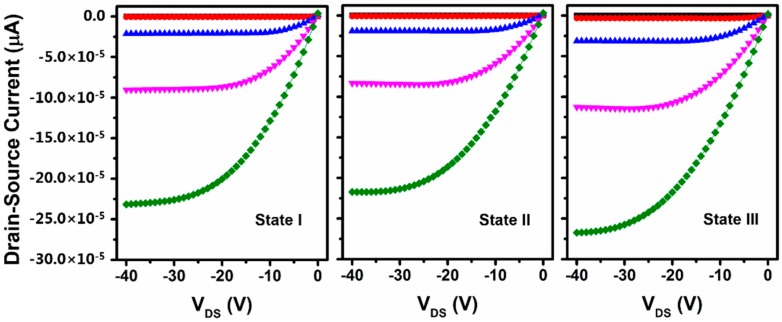
Output characteristics (*V_G_* = 0 to −40 V, with a −10 V step) of pentacene-based OFETs with different gate biases and NH_3_ concentrations. State I: gate bias was 0 V and no NH_3_; State II: gate bias was 0 V and 10 ppm NH_3_; State III: gate bias was −40 V and 10 ppm NH_3_.

NH_3_ is a polar gas and, when it absorbs into *p*-type organic semiconductors, it will act as a hole trap and result in a decrease of *I_DS_* [6]. We applied different gate bias voltages when the OFETs were exposed to different conditions, the output curves were shown in Figure 5. When the devices were exposed to NH_3_ circumstances with 10 ppm under 0 V for 5 min, NH_3_ molecules were absorbed and distributed onto the semiconductor, as well as into the channel. However, due to the additional hole charge induced by hydroxyl groups, the output current decreased slightly (as shown in Figure 5, state II). When the gate bias was −40 V, the hydroxyl groups began to polarize as before. Since the NH_3_ molecules also possessed a permanent dipole moment, all of the NH_3_ dipoles present at the interfaces were also polarized immediately and caused a huge dipole moment at the interface. This dipole field interacted with the polar hydroxyl groups through the nonpolar PMMA layer. As a result, the induced charges at the accumulation layer were further enhanced in the presence of NH_3_ as compared to atmosphere conditions where there were no NH_3_ molecules in the devices. Therefore, the output current did not decrease, but increased as shown in Figure 5.

From the above analysis, the schematic of hydroxyl groups in the dielectric layer has been proposed that can be applied to a *p*-type organic semiconductor. Thus, we assume that, if the hydroxyl groups could be aligned under negative voltage (O atoms upside), they should be able to be reversed under positive voltage (H atoms upside) to induce electron-charges at the semiconductor/dielectric interface.

**Figure 6:**
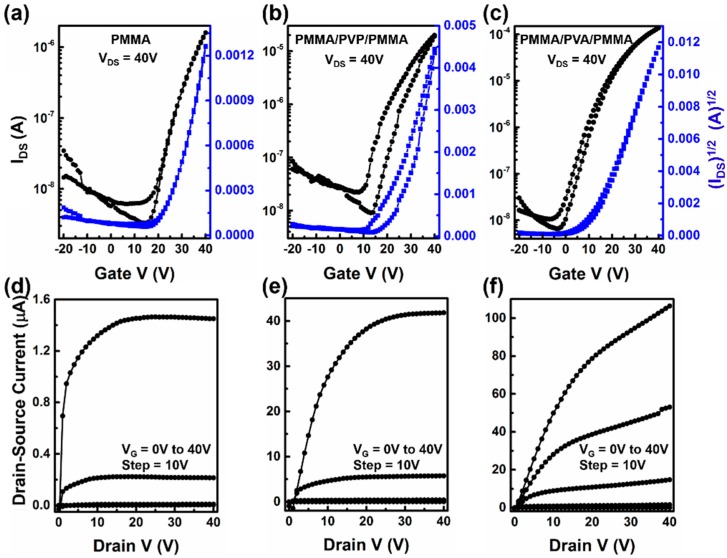
Transfer (*V_DS_* = 40 V) and output (*V_G_* = 0 to 40 V, with a 10 V step) characteristics of PTCDI-C8-based OFETs with different dielectric structures. (**a**,**d**) PMMA; (**b**,**e**) PMMA/PVP/PMMA; (**c**,**f**) PMMA/PVA/PMMA.

To rule out that the effect does not depend much on the organic semiconductor materials used for the fabrication of the devices, we have performed the same measurements on OTFTs fabricated using a conventional *n*-type organic semiconductor, PTCDI-C8 [33]. Figure 6 depicts the representative *n*-type transfer (−20 V to 40 V) and output plots of the OFETs with different dielectric structures prepared from PTCDI-C8. All of the characteristics including *µ*, *I_on_*/*I_off_*, *V_T_*, and *SS* of different devices are presented in Table 2. Not unexpectedly, OFETs containing polar polymer layers exhibited an obvious improvement as the *p*-type devices. When the gate voltage was switched up, the hydroxyl groups began to align and polarize as aforementioned. Since the gate bias was 40 V, the induced charges in accumulation layer were electrons. Hence, the maximum improvements of parameters increase from 1.5 to 108 μA, 0.03 cm^2^V^−1^s^−1^ to 0.43 cm^2^V^−1^s^−1^, and 0.5 × 10^3^ to 2.1 × 10^4^ of *I_DS_*, *μ*, and *I_on_*/*I_off_*, respectively. This result showed that the observed effect was the intrinsic behavior of the dielectric system, and did not rely much on the organic semiconductor materials of OFETs. Performance enhancement in OFETs based on applied gate bias was essentially due to the polarization of the polar dielectric layer in the presence of the gate field.

Note that α-6T was reported as an excellent templating material for both ambipolar OFETs and light-emitting FETs [34,35]; P3HT always acted as a model conjugated polymer due to its high hole mobility, solution processibility, and potential to self-assemble during processing [36]. To further verify the abovementioned assumptions, the organic semiconductors of α-6T and P3HT were used as active layers to fabricate OFETs containing a PVA dielectric layer. Their transfer and output curves are shown in Figures S2 and S3. The *µ*, *I_on_/I_off_*, *V_T_*, and SS of different devices are listed in Table S1. It can be observed that all the parameters have significant improvements, which is similar to pentacene devices. In particular, the µ calculated from curve shown in Figure S2c reached ~0.08 cm^2^V^−1^s^−1^, which was on the same order as that obtained from single-crystal devices [37]. As shown in Figure S3, the *V_T_* of P3HT-OFETs shifted from −6 V to −1 V when using PVA as the sandwiched dielectric layer. This phenomenon indicates that this kind of dielectric component has potential to realize solution-processed, low-voltage, and high field-effect mobility OFETs.

## 3. Materials and Methods

### 3.1. Device Preparation

The OFETs were fabricated on indium tin oxide glass substrates. Prior to the deposition of dielectric layers, the substrates were successively ultrasonically cleaned in acetone, deionized water, and isopropyl alcohol. PVA (Mw ~ 146 k–186 kDa, 99+% hydrolyzed), PMMA (average Mw ~ 120 kDa), and PVP (average Mw ~ 25 kDa) were dissolved in deionized water (40 mg/mL), anisole (50 mg/mL and 100 mg/mL), and butyl acetate (40 mg/mL), respectively. PMMA (50 mg/mL) was spin-coated on ITO substrate at 2000 rpm for 60 s (about 150 nm) and baked in an oven at 90 °C for 1 h, PVA was spin-coated on PMMA at 3000 rpm for 60 s (about 150 nm), and PVP was spin-coated on PMMA at 2000 rpm for 60 s (about 150 nm), PMMA (50 mg/mL) on the top layer was spin-coated on at 3000 rpm for 60 s (about 100 nm). For comparison, a single layer of PMMA (100 mg/mL) was spin-coated on ITO substrate at 1500 rpm for 60 s (about 400 nm) and baked in an oven at 90 °C for 1 h. Subsequently, pentacene, PTCDI-C8, and α-6T were thermally deposited under 2 × 10^−4^ Pa at the rate of 0.2–0.3 Å/s. P3HT (8 mg/mL dissolved in dichlorobenzene) was spin-cast onto the dielectric layer using an on-the-fly-dispensing spin-coating approach (2000 rpm), in which the solution was dispensed when the spin-coater motor was already operating at fixed rotation speed [38]. The residual solvent was removed by drying the samples in a vacuum oven at 120 °C for 20 min. All of the materials were purchased from Sigma-Aldrich (Shanghai, China) and used without further purification. Finally, the source and drain electrodes of 50 nm gold (Au) were thermally deposited on the blended film and patterned with a shadow mask. The length and width of the channels were 100 μm and 1 cm, respectively.

### 3.2. Device Characterization

The electrical characteristics of all the devices were measured with a Keithley 4200-SCS Source Measure Unit (Tektronix, Shanghai, China) under ambient conditions. The field-effect mobility of devices was extracted in the saturation regime from the highest slope of |*I_DS_*|^1/2^ vs. *V_G_* plots by using Equation (2):

(2) $$I_{DS}=(\frac{WC_{i}}{2L})\mu {\left(V_{G}-V_{T}\right)}^{2}$$

where *L* and *W* are the channel length and width, respectively. *C_i_* is the capacitance (per unit area) of the dielectric, *V_G_* is the gate voltage, and *I_DS_* is the drain-source current. In this work, the *C_i_* of PMMA, PMMA/PVA/PMMA, and PMMA/PVP/PMMA were 6.3 nF/cm^2^, 11.2 nF/cm^2^, and 9.4 nF/cm^2^, respectively (see in Figure S4). The morphologies of pentacene films were analyzed by atomic force microscopy (AFM, Agilent, Shanghai, China) images. The thickness of the dielectrics was measured by a Dektak 150 stylus profiler (Veeco, Shanghai, China).

The NH_3_ test of OFET was stored in an airtight test chamber (approximately 0.02 L). Dry air and 500 ppm standard NH_3_ gases (anhydrous) were purchased from Sichuan Tianyi Science and Technology Co. (Chengdu, China), and a mixture with the appropriate concentration was introduced into the test chamber by a mass flow controller.

## 4. Conclusions

In summary, the enhanced performance with the high field-effect mobility, low voltage, and high on/off ratio of polymer-dielectric OFETs was obtained when a polar polymer dielectric containing hydroxyl groups was sandwiched between hydrophobic and nonpolar layers. Contrary to the existing notion that the hydroxyl groups at the dielectric/semiconductor interface must be eliminated to weaken their trapping effects on charge carriers, we insulated the hydroxyl groups away from accumulation layer and align them by applied gate voltage. As a result, an additional field along the gate-field direction appeared due to the polarization of oriented hydroxyl groups, which induced extra charge in accumulation layer. These devices showed a dramatic performance improvement of several parameters, such as field-effect mobility, threshold voltage, on/off ratio, and drain current. A schematic based on the obtained result was proposed to illustrate this phenomenon. Additionally, this schematic was verified to be universal for both *n*-type and *p*-type organic semiconductors as active layers of OFETs. This work proposes a reference that high-performance polymer-dielectric OFETs can be realized by a simple trilayer dielectric system containing a polar polymer as a sandwiched layer and a nonpolar outside. The high-performance OFETs, with lower fabrication cost, enable large-scale and flexibility to become feasible.

## Figures and Tables

**Table 1:** Characteristics of pentacene-based OFETs with different dielectric structure.

Dielectric Structure	*μ* (cm^2^V^−1^s^−1^)	*V_T_* (V)	*SS* (V/dec)	*On*/*Off Ratio*
PMMA	0.17 ± 0.005	−16 ± 4.0	5.0 ± 0.2	2.4 × 10^3^
PMMA/PVP/PMMA	0.45 ± 0.200	−10 ± 2.0	3.8 ± 0.2	1.1 × 10^4^
PMMA/PVA/PMMA	0.51 ± 0.150	−2 ± 0.5	1.5 ± 0.2	3.5 × 10^5^

**Table 2:** Characteristics of PTCDI-C8-based OFETs with different dielectric structures.

Dielectric Structure	*μ* (cm^2^V^−1^s^−1^)	*V_T_* (V)	*SS* (V/dec)	*On*/*Off Ratio*
PMMA	0.03 ± 0.01	25 ± 5	6.0 ± 0.2	5.0 × 10^2^
PMMA/PVP/PMMA	0.11 ± 0.02	18 ± 4	5 ± 0.2	2.1 × 10^3^
PMMA/PVA/PMMA	0.43 ± 0.11	10 ± 2	4 ± 0.2	2.1 × 10^4^

## Supplementary Materials

The following are available online at www.mdpi.com/1996-1944/9/7/545/s1.

## References

[1] Gelinck G., Heremans P., Nomoto K., Anthopoulos T.D. (2010). Organic transistors in optical displays and microelectronic applications. Adv. Mater..

[2] Lee J., Kim D.H., Kim J.Y., Yoo B., Chung J.W., Park J.I., Lee B.L., Jung J.Y., Park J.S., Koo B. (2013). Reliable and uniform thin-film transistor arrays based on inkjet-printed polymer semiconductors for full color reflective displays. Adv. Mater..

[3] Fan C.L., Lin W.C., Chang H.S., Lin Y.Z., Huang B.R. (2016). Effects of the F4TCNQ-doped pentacene interlayers on performance improvement of top-contact pentacene-based organic thin-film transistors. Materials.

[4] Fujisaki Y., Koga H., Nakajima Y., Nakata M., Tsuji H., Yamamoto T., Kurita T., Nogi M., Shimidzu N. (2014). Transparent nanopaper-based flexible organic thin-film transistor array. Adv. Funct. Mater..

[5] Hyun W.J., Secor E.B., Rojas G.A., Hersam M.C., Francis L.F., Frisbie C.D. (2015). All-printed, foldable organic thin-film transistors on glassine paper. Adv. Mater..

[6] Yu J., Yu X., Zhang L., Zeng H. (2012). Ammonia gas sensor based on pentacene organic field-effect transistor. Sens. Actuators B Chem..

[7] Han S., Zhuang X., Shi W., Yang X., Li L., Yu J. (2016). Poly (3-hexylthiophene)/polystyrene (P3HT/PS) blends based organic field-effect transistor ammonia gas sensor. Sens. Actuators B Chem..

[8] Shi W., Yu X., Zheng Y., Yu J. (2016). DNA based chemical sensor for the detection of nitrogen dioxide enabled by organic field-effect transistor. Sens. Actuators B-Chem..

[9] Minamiki T., Minami T., Kurita R., Niwa O., Wakida S., Fukuda K., Kumaki D., Tokito S. (2014). A label-free immunosensor for IgG based on an extended-gate type organic field effect transistor. Materials.

[10] Ortiz R.P., Facchetti A., Marks T.J. (2010). High-k organic, inorganic, and hybrid dielectrics for low-voltage organic field-effect transistors. Chem. Rev..

[11] Li Q., Chen L., Gadinski M.R., Zhang S., Zhang G., Li H., Haque A., Chen L., Jackson T., Wang Q. (2015). Flexible high-temperature dielectric materials from polymer nanocomposites. Nature.

[12] Pecunia V., Banger K., Sirringhaus H. (2015). High-performance solution-processed amorphous-oxide-semiconductor TFTs with organic polymeric gate dielectrics. Adv. Electron. Mater..

[13] Reyes-Martinez M.A., Crosby A.J., Briseno A.L. (2015). Rubrene crystal field-effect mobility modulation via conducting channel wrinkling. Nat. Commun..

[14] Yuan Y., Giri G., Ayzner A.L., Zoombelt A.P., Mannsfeld S.C.B., Chen J., Nordlund D., Toney M.F., Huang J., Bao Z. (2015). Ultra-high mobility transparent organic thin film transistors grown by an off-centre spin-coating method. Nat. Commun..

[15] Wang S., Fabiano S., Himmelberger S., Puzinas S., Crispin X., Salleo A., Berggren M. (2015). Experimental evidence that short-range intermolecular aggregation is sufficient for efficient charge transport in conjugated polymers. Proc. Natl. Acad. Sci. USA.

[16] Han S., Yu X., Shi W., Zhuang X., Yu J. (2015). Solvent-dependent electrical properties improvement of organic field-effect transistor based on disordered conjugated polymer/insulator blends. Org. Electron..

[17] Wong C.Y., Cotts B.L., Wu H., Ginsberg N.S. (2015). Exciton dynamics reveal aggregates with intermolecular order at hidden interfaces in solution-cast organic semiconducting film. Nat. Commun..

[18] Yagi M., Ito N., Kawasaki M., Shimomura T. (2016). Semiconducting properties of p- and n-type organic nanofiber/poly(methyl methacrylate) composite films for film rectifier. Synth. Met..

[19] Zhang Y., Qiao J., Gao S., Hu F., He D., Wu B., Yang Z., Xu B., Li Y., Shi Y. (2016). Probing carrier transport and structure-property relationship of highly ordered organic semiconductors at the two-dimensional limit. Phys. Rev. Lett..

[20] Tsai T.D., Chang J.W., Wen T.C., Guo T.F. (2013). Manipulating the hysteresis in poly(vinyl alcohol)-dielectric organic field-effect transistors toward memory elements. Adv. Funct. Mater..

[21] Lee S., Koo B., Shin J., Lee E., Park H., Kim H. (2006). Effect of hydroxyl groups in polymeric dielectrics on organic transistor performance. Appl. Phys. Lett..

[22] Orgiu E., Locci S., Fraboni B., Scavetta E., Lugli P., Bonfiglio A. (2011). Analysis of the hysteresis in organic thin-film transistors with polymeric gate dielectric. Org. Electron..

[23] Hwang D.K., Lee K., Kim J.H., Im S., Park J.H., Kim E. (2006). Comparative studies on the stability of polymer versus SiO_2_ gate dielectrics for pentacene thin-film transistors. Appl. Phys. Lett..

[24] Egginger M., Vladu M.I., Schwödiauer R., Tanda A., Frischauf I., Bauer S., Sariciftci N.S. (2008). Mobile ionic impurities in poly(vinyl alcohol) gate dielectric: Possible source of the hysteresis in organic field-effect transistors. Adv. Mater..

[25] Ukah N.B., Senanayak S.P., Adil D., Knotts G., Granstrom J., Narayan K.S., Guha S. (2013). Enhanced mobility and environmental stability in all organic field-effect transistors: The role of high dipole moment solvent. J. Polym. Sci. Part B Polym. Phys..

[26] Ukah N.B., Granstrom J., Gari R.R.S., King G.M., Guha S. (2011). Low-operating voltage and stable organic field-effect transistors with poly (methyl methacrylate) gate dielectric solution deposited from a high dipole moment solvent. Appl. Phys. Lett..

[27] Subbarao N.V.V., Gedda M., Iyer P.K., Goswami D.K. (2015). Enhanced environmental stability induced by effective polarization of a polar dielectric layer in a trilayer dielectric system of organic field-effect transistors: A quantitative study. ACS Appl. Mater. Interfaces.

[28] Kim J., Park C.J., Yi G., Choi M.S., Park S.K. (2015). Low-temperature solution-processed gate dielectrics for high-performance organic thin film transistors. Materials.

[29] Arias A.C., MacKenzie J.D., McCulloch I., Rivnay J., Salleo A. (2010). Materials and applications for large area electronics: Solution-based approaches. Chem. Rev..

[30] Pauli M., Zschieschang U., Barcelos I.D., Klauk H., Malachias A. (2016). Tailoring the dielectric layer structure for enhanced carrier mobility in organic transistors: The use of hybrid inorganic/organic multilayer dielectrics. Adv. Electron. Mater..

[31] Klauk H. (2010). Organic thin-film transistors. Chem. Soc. Rev..

[32] Huang W., Yu J.S., Yu X.G., Shi W. (2013). Polymer dielectric layer functionality in organic field-effect transistor based ammonia gas sensor. Org. Electron..

[33] Kola S., Sinha J., Katz H.E. (2012). Organic transistors in the new decade: Toward n-channel, printed, and stabilized devices. J. Polym. Sci. Part B Polym. Phys..

[34] Yang J., Yan D., Jones T.S. (2015). Molecular template growth and its applications in organic electronics and optoelectronics. Chem. Rev..

[35] Pham S.T., Tada H. (2014). Magnetoluminescence of light-emitting field-effect transistors based on alpha sexithiophene. Appl. Phys. Lett..

[36] Lee C.S., Yin W., Holt A.P., Sangoro J.R., Sokolov A.P., Dadmun M.D. (2015). Rapid and facile formation of P3HT organogels via spin coating: Tuning functional properties of organic electronic thin films. Adv. Funct. Mater..

[37] Horowitz G., Garnier F., Yassar A., Hajlaoui R., Koukl F. (1996). Field-effect transistor made with a sexithiophene single crystal. Adv. Mater..

[38] Zhang F., Di C., Berdunov N., Hu Y., Hu Y., Gao X., Meng Q., Sirringhaus H., Zhu D. (2013). Ultrathin film organic transistors: Precise control of semiconductor thickness via spin-coating. Adv. Mater..

