# Supplementary material for: Tailoring the Dielectric Layer Structure for Enhanced Performance of Organic Field-Effect Transistors: The Use of a Sandwiched Polar Dielectric Layer
Source: Materials (Basel). 2016 Jul 7;9(7):545. doi: 10.3390/ma9070545 (PMC5456942; doi:10.3390/ma9070545)
Supplement: Supplementary file 1 [file materials-09-00545-s001.pdf]

Supplementary Materials: Tailoring the Dielectric Layer Structure for Enhanced Performance of Organic Field-Effect Transistors: The Use of a Sandwiched Polar Dielectric Layer

Shijiao Han, Xin Yang, Xinming Zhuang, Junsheng Yu and Lu Li

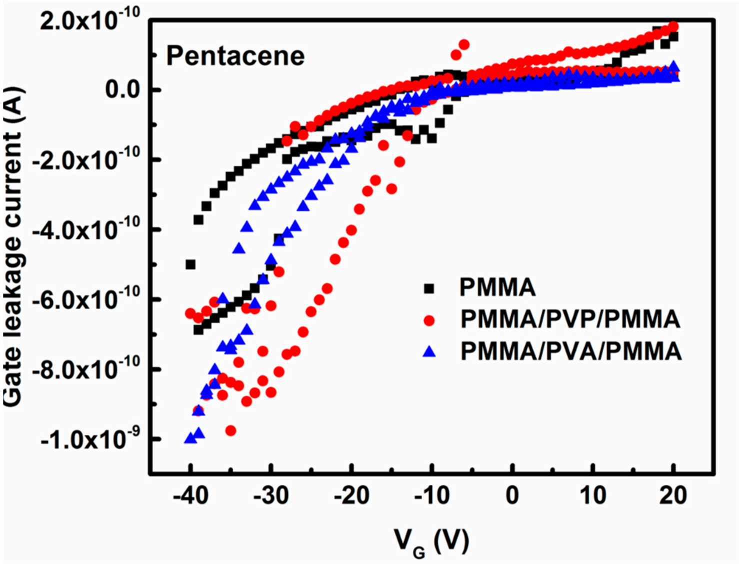

Figure S1. Gate leakage current of pentacene-based OFETs with different dielectric structures.

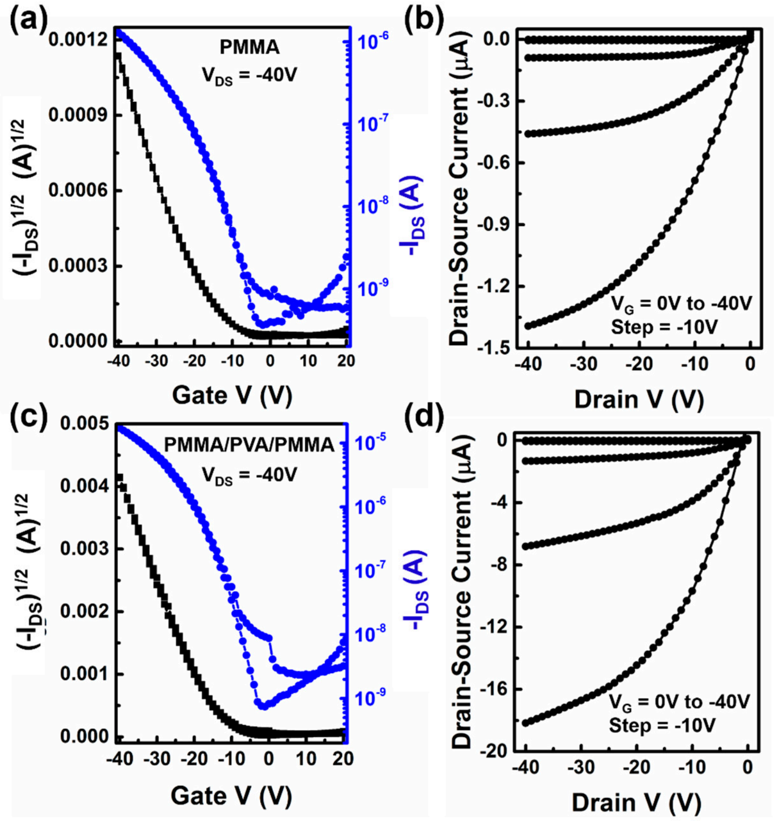

Figure S2. Typical transfer (V_{DS} = −40 V) and output (V_G = 0 to −40 V, with a −10 V step) characteristics of α-6T-based OFETs with different dielectric structures. (**a**,**b**) PMMA; (**c**,**d**) PMMA/PVA/PMMA.

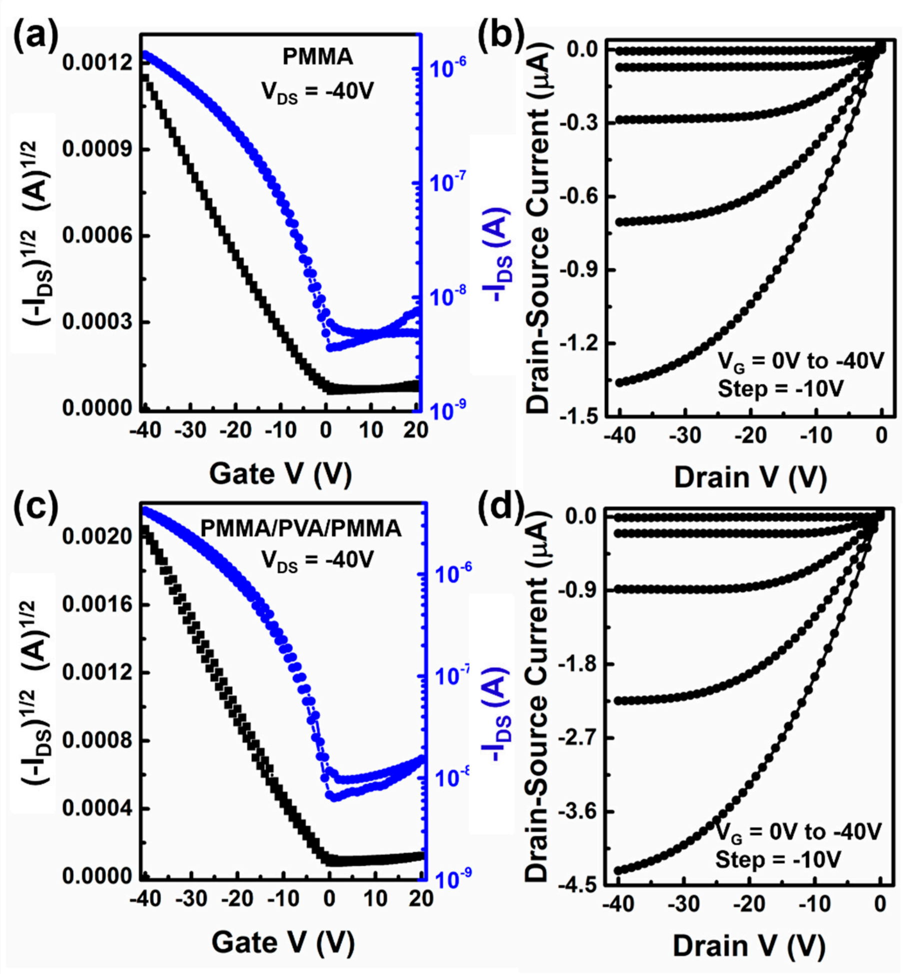

Figure S3. Typical transfer (V_{DS} = −40 V) and output (V_G = 0 to −40 V, with a −10 V step) characteristics of P3HT-based OFETs with different dielectric structures. (**a**,**b**) PMMA; (**c**,**d**) PMMA/PVA/PMMA.

Table S1. Characteristics of OFETs with a PVA dielectric middle layer.

Organic Semiconductor	Dielectric Structure	μ (cm^{2}V^{-1}s^{-1})	V_T (V)	*SS* (V/dec)	*On*/*Off* Ratio
α6T	PMMA	0.01 ± 0.002	−18 ± 4.0	6 ± 0.5	3.6 × 10^3
	PMMA/PVA/PMMA	0.08 ± 0.010	−14 ± 2.0	3 ± 0.5	2.3 × 10^4
P3HT	PMMA	0.004 ± 0.001	−6 ± 2.0	7 ± 0.5	4.0 × 10^2
	PMMA/PVA/PMMA	0.011 ± 0.003	−1 ± 0.5	5 ± 0.2	7.0 × 10^2

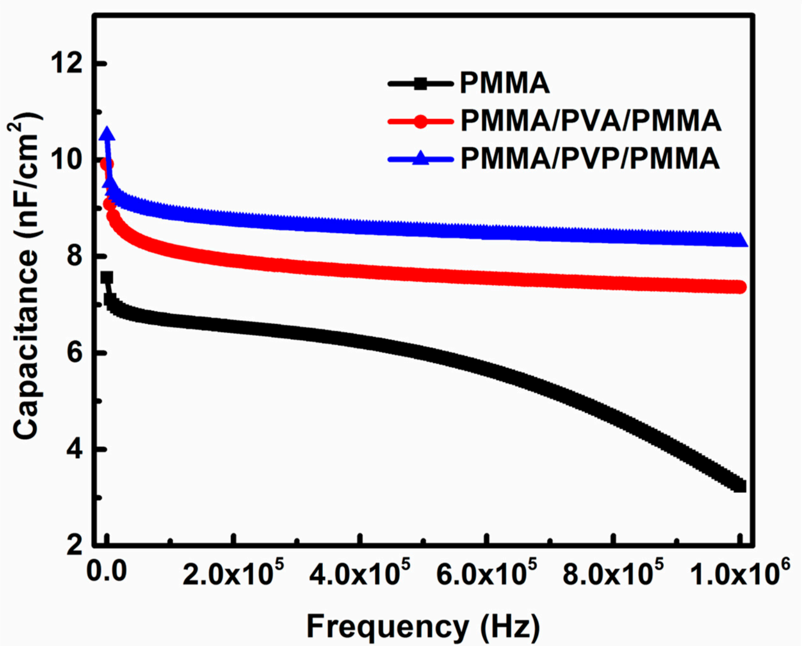

Figure S4. Dielectric constant (per unit area) vs. frequency plots of different dielectric structures used for OTFTs.
